# Validation of Perceived Mental Fatigability Using the Chinese Version of the Pittsburgh Fatigability Scale

**DOI:** 10.1093/geroni/igab046.2051

**Published:** 2021-12-17

**Authors:** Yixin Hu, Hangming Zhang, Weihao Xu, siyue wang, Woei-Nan Bair, Li Fan, Yujia (Susanna) Qiao, Nancy W Glynn

**Affiliations:** 1 Chinese PLA General Hospital, Beijing, Beijing, China (People's Republic); 2 Peking university shougang hospital, ., Beijing, China (People's Republic); 3 Peking university School of Public Health, ., Beijing, China (People's Republic); 4 University of the Sciences in Philadelphia, Philadelphia, Pennsylvania, United States; 5 University of Pittsburgh, University of Pittsburgh, Pennsylvania, United States; 6 University of Pittsburgh Graduate School of Public Health, Pittsburgh, Pennsylvania, United States

## Abstract

Background: Recently we validated the simplified-Chinese version of the Pittsburgh Fatigability Scale (PFS) Physical subscale. Next step is to validate the PFS Mental subscale in order to introduce a reliable measure of perceived mental fatigability among Chinese community-dwelling older adults. Methods: This cross-sectional study was conducted in an urban community in Beijing. Internal consistency of the PFS Mental subscale was evaluated by Cronbach’s alpha. The participants were divided in half to evaluate the factor structure validity by exploratory factor analyses and confirmatory factor analysis. Convergent validity and discriminant validity were evaluated against cognitive function (assessed by MOCA) and global fatigue from FRAIL Scale. Results: Our study included 370 participants (mean=83.8 years). The simplified-Chinese version of PFS Mental subscale showed strong internal consistency (total Cronbach’s alpha=0.82, each items Cronbach’s alpha ranged from 0.78 – 0.83). The results of exploratory factor analysis showed all 10 items loaded on two factors: moderate to high and low intensity activities, which explained 60.8% of the total variance. Confirmatory factor analysis showed fit indices: SRMSR = 0.090, RMSEA = 0.120, CFI = 0.89. PFS Mental scores demonstrated moderate concurrent and construct validity against cognitive function (r = －0.24, P<.001). Additionally, the PFS Mental subscale had strong convergent validity, discriminating according to established cognitive impairment or FRAIL Scale fatigue testing cut points, with differences in PFS Mental scores ranging from 3.2 to 8.4 points. Conclusions: The PFS Mental subscale simplified-Chinese version is a valid tool to assess perceived mental fatigability in Chinese-speaking older adults.

